# Performance Evaluation of Stabilized Soils with Selected Common Waste Materials of Rice Husk Ash, Steel Slag and Iron Tailing Powder

**DOI:** 10.3390/ma18020346

**Published:** 2025-01-14

**Authors:** Degou Cai, Mingzhe Ouyang, Xinyu Bao, Qianli Zhang, Zongqi Bi, Hongye Yan, Shimin Li, Yuefeng Shi

**Affiliations:** 1 Railway Engineering Research Institute, China Academy of Railway Sciences Corporation Limited, Beijing 100081, China; 2 School of Soil and Water Conservation, Beijing Forestry University, Beijing 100083, China; 3 School of Civil Engineering, Shijiazhuang Tiedao University, Shijiazhuang 050043, China

**Keywords:** soil stabilization, common waste materials, hydration degree, cementitious materials

## Abstract

Soil stabilization technology has been applied for a long time in the infrastructure construction field. Currently, the use of waste materials as stabilizer is growing in attention, because it promises to develop green and high-performance soil stabilization efficiency. In this work, three common waste materials, including rice husk ash (RHA), steel slag (SS) and iron tailing (IT) powder, were selected and synergistically utilized with cement to prepare stabilized soil. The mechanical strength, hydration degree and microstructure of the stabilized soil samples were tested. The experimental results showed that the mechanical strengths of the samples were improved as the cement content increased. To be specific, RHA-blended samples exhibited the lowest strengths compared with those incorporating SS and IT, indicating the poor effect of RHA on stimulating strength improvement. Moreover, SS and IT showed a much more significant effect on enhancing the mechanical strength for the stabilized soil samples, and the strength increasing rates can reach up to 60% compared to the reference batch. In addition, microstructural analysis results further verified the benefits of cement and waste materials on improving the performance of stabilized soil samples, as the hydration reaction and pore structure were proven to be improved with the aid of waste materials. This work gives insights into environmentally friendly road construction with high utilization of selected common wastes.

## 1. Introduction

Road engineering is a pivotal link in the development of the economy and society, and can be regarded as foundational [1,2]. The infrastructure construction of road engineering consumes a large amount of road construction materials, including soil stabilizer, cement, cementitious materials, soil, rock, etc. [3,4,5]. With the continuously increasing demand for road construction, consumption of the related raw materials is also significantly rising, while the amount of traditional rock materials is decreasing because mines are shutting down for the sake of environmental policies. In this context, finding a suitable replacement material for rock is essential. At the same time, a large amount of waste soil and silt can be produced during the tunnel digging and infrastructure construction process, and the disposal of these materials is still a tough problem. For now, the waste soil and silt have occupied many farmlands and caused a series of environmental pollution risks. Under these circumstances, an effective utilization of waste soil and silt for the construction of road engineering is a key issue to be addressed [6,7].

Soil solidification technology is an efficient method to solve the abovementioned issue, because this method can increase the mechanical strength of the soil with various requirements [8,9,10]. In general, the most popular soil stabilizers are cement, limestone powder and fly ash, while these materials exhibited certain limitations, including the high carbon trace of cement, the low early strength of limestone powder, fly ash and blended soil and so on [6,7,11,12]. Moreover, the soil stabilized by cement, limestone powder and fly ash showed low acceptability to saline soil and expansive soil. Therefore, a new stabilizer for soil needs to be investigated to achieve the goal of lowering carbon emissions alongside reliable performance.

The use of industrial or agricultural byproducts can be effective in soil stabilization technology, among which, the rice husk ash (RHA), steel slag (SS) and iron tailing (IT) show promise [13,14,15]. RHA is the high temperature calcination product of rice husk, which can be obtained through the rice milling process. It was reported that the annual production of paddy reached up to above 750 million tons, in which the rice husk took up above 20% [16,17]. The high production of rice husk contributed to the biomass’ generated energy through various treatment processes, and the RHA remained after the treatment. RHA is always regarded as a common waste material and discarded for landfill engineering, while this disposal method contaminates the surrounding soil with a heavy environmental burden. Considering the benefits of RHA with good pozzolanic reactivity, it is possible to use RHA as the soil stabilizer with its low cement content [18]. Moreover, SS is a typical by-product from the steel-manufacturing process [19,20]. As reported, the discarded amount of SS in China is increasing at the rate of 100 million tons per year, and the processing rate of SS is still lower than 40%. If SS is not rationally treated, it occupies a significant area of land and pollutes the environment. To be more precise, the composition of SS is similar to that of cement, making it a promising substitution for cement as soil stabilizer [21,22]. Furthermore, the rapid development of industrialization increases the production of waste tailings, which is now gradually becoming a major concern with respect to environmental and resource management [23,24]. It should be noted that the annual production of tailings in China reaches above 15 billion tons. Among the tailings, the production of IT can take up above 35%, while the processing efficiency of IT is lower than 20%, which is even lower than that of SS. Under these circumstances, the efficient treatment of IT is in great demand to realize the tailing accumulation [25,26]. Similar to the two abovementioned materials, IT is also considered a promising material for application as soil stabilizer in road construction, because the hydration reactivity of IT can facilitate the reaction inside the sample [27,28,29]. In conclusion, RHA, SS and IT may be useful to serve as soil stabilizers for road construction because these materials all exhibit a certain degree of hydration reactivity, but the basic effects of the above materials still need to be further investigated, aiming to better understand the specific influence of various stabilizers on the performance of soil. Also, the combined effects of common waste and cement on the performance of stabilized soil should also be demonstrated, because the hydration reaction of cement may be more significant in a cementitious material system. It should also be noted that the utilization of RHA, SS and IT may be reported in some of the literature, but was still very rare compared to the traditional SCMs, such as fly ash, silica fume and slag, etc. Moreover, a direct link among the three materials in the performance of stabilized soil was also lacked. In this context, research regarding the effect of RHA, SS and IT as soil stabilizers with various contents, and the combined effect of the above materials with the cement should be conducted.

In this work, three selected industrial or agricultural by-products (RHA, SS and IT) were synergistically utilized as the stabilizer with low cement content to stabilize soil, aiming to effectively improve the performance of stabilized soil with remarkable environmental benefits. The 28-day unconfined compressive strengths of the samples were measured to evaluate the mechanical strength of the soil sample with various cement contents and common waste materials. Thermogravimetric (TG) analysis was conducted to evaluate the degree of hydration inside the soil samples, and nitrogen adsorption analysis was conducted to measure the specific surface area for the samples based on the Brunauer–Emmett–Teller (BET) theory, and the pore structure information of the soil samples was further clarified. The obtained microstructural results were used to reflect the specific effects of the stabilizers and cement on soil stabilization efficiency.

## 2. Materials and Methods

### 2.1. Raw Materials for Preparing Stabilized Soil

The soil that was used was the soft soil collected from a construction site with a depth under 2 m, and the physical properties of the soil are listed in Table 1. Moreover, the soil was dried in a drying oven at 60 °C until the weight of soil became constant, to ensure stable physical and chemical properties, and the soil was ground to pass through the sieve of 4.75 mm for further use. Moreover, Ordinary Portland cement with a strength grade of 42.5 MPa (OPC 42.5) was used as the main cementitious material. The RHA, SS and IT were respectively purchased from the treatment companies for the raw materials, and the detailed chemical compositions of the cement and stabilizers are listed in Table 2.

### 2.2. Stabilized Soil Sample Preparation Method

In this work, the content of cement or stabilizers indicated the weight percentage of the raw materials in the dried soil. The cement contents were set to be 4%, 5% and 6%, and the contents of the stabilizers were determined to vary from 0% to 8% (taking 2% as the first step). According to Specification JTG E51-2009 [30], the soil, cement and stabilizers were mixed first, then the water was added into the soil sample, and the mixture was further mixed in the stirring pot. The degree of compaction for the soil sample was set to 95% to ensure the basic mechanical strength, and the fresh sample was cast into the cylinder mold with the volumetric size of 50 mm × 50 mm (with a diameter of 50 mm and height of 50 mm). The sample was demolded after the initial 4 h curing, and the samples were sealed for further curing. The sample was cured at the standard curing condition (temperature: 20 ± 2 °C, relative humidity > 95%) to the pre-set curing age.

### 2.3. Mechanical Strength Test Method

The 28-day unconfined compressive strengths of the samples were measured according to JTG D40-2007 [31]. During the test process, the velocity was set to be 1 mm/min. To be more specific, the samples were taken out from the curing room one day in advance and the samples were immersed in the water, and the test was conducted after one day of water immersion. Three samples were used for a mechanical strength test, and the average value was taken as representative.

### 2.4. TG Analysis

The TG test was conducted for the hydration degree analysis of the stabilized soil samples, and the used instrument was STA449F3, produced by Netzsch Company (Selb, Germany). The samples were initially broken into small pieces and immersed in absolute ethyl alcohol to terminate the hydration reaction of the cementitious materials, then the small blocks were dried at 60 °C to eliminate influence from the water. The heating rate during the TG test was set to be 10 °C per min, and the N_2_ atmosphere served as the protection gas during the test.

### 2.5. Specific Surface Area and Pore Structure Analysis

The micropore structure analyses of the stabilized soil samples were further conducted by the BET method through the instrument of ASAP 2020, Micromeritics company (Norcross, GA, USA), because this method reflects the specific surface area inside the sample and reflects the size distribution of the pores in nano scale. Before the test, the sample was degassed in the vacuum environment at a low temperature for 8 h, and nitrogen served as the adsorbate.

## 3. Results and Discussion

### 3.1. Unconfined Compressive Strength of Stabilized Soil Samples

Mechanical strength is the most significant indicator for the stabilized soil sample, because it directly reflects the performance of the soil, and the strength can determine the feasibility of the soil sample in actual road engineering. In this work, the 28-day unconfined compressive strengths of the stabilized soil samples were tested. The cement contents, the selected common waste materials and the content of the waste materials were changed as the variables to elucidate the feasibility, which is ordinary, as reported in the literature [32,33,34].

**Figure 1:**
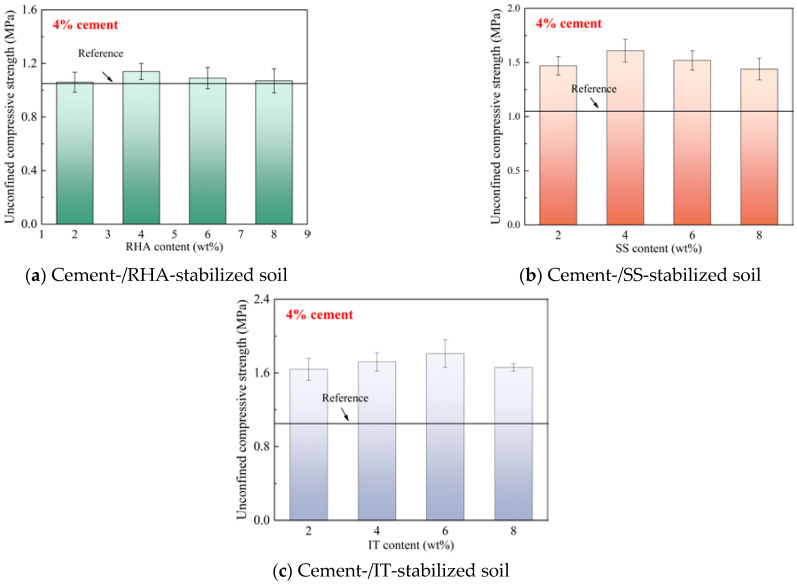
28-day unconfined compressive strength of stabilized soil with 4% cement content.

The 28-day unconfined compressive strengths of the soil samples with 4% cement content and various common wastes contents are depicted in Figure 1. It was found that the incorporation of selected common wastes showed various effects on the 28-day unconfined compressive strengths of the samples. To be specific, the inclusion of RHA exhibited little influence on the mechanical strength of the stabilized soil, the highest compressive strength was found in the sample containing 4% RHA with the value lower than 1.2 MPa, and the strengths of the samples containing 2% and 8% RHA were close to those of the reference batch. However, SS and IT largely improve the 28-day unconfined compressive strengths of the samples, and the compressive strengths of SS-blended samples were around 1.5 MPa, with the highest value of 1.61 MPa. For the IT-blended samples, the compressive strength reached up to 1.81 MPa when the IT content was 6%. The higher compressive strength in SS- and IT-blended samples can be attributed to the more intense reaction activity of these materials in the matrix compared to that of RHA with a much lower reaction activity.

**Figure 2:**
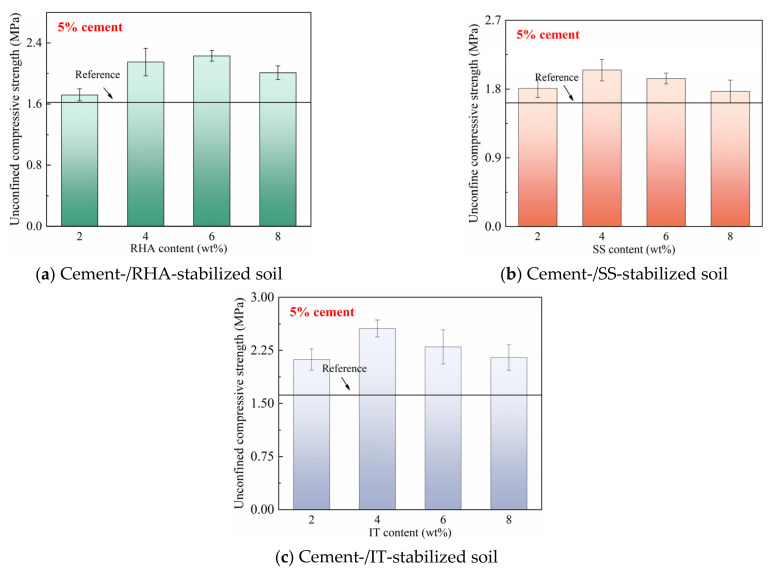
28-day unconfined compressive strength of stabilized soil with 5% cement content.

The mechanical strength test results at a cement content of 5% are shown in Figure 2. It was found that increasing cement contents improved the unconfined compressive strength of the samples, because the more intense hydration reaction of cement inside the matrix and the increased cement content may even further activate the reaction of the waste materials. Moreover, the RHA exhibited a more significant effect on improving the compressive strength of the samples. Specifically, when the content of RHA was 6%, the compressive strength of the sample reached up to 2.23 MPa, gaining an increase of 29% compared to the reference sample. As for the samples containing SS and IT, the compressive strengths were also accordingly increased, and the optimal contents of SS and IT were determined to be 4%, based on the results. The intense hydration reaction of cement inside the samples may facilitate the reaction of waste materials, thus resulting in higher compressive strength.

**Figure 3:**
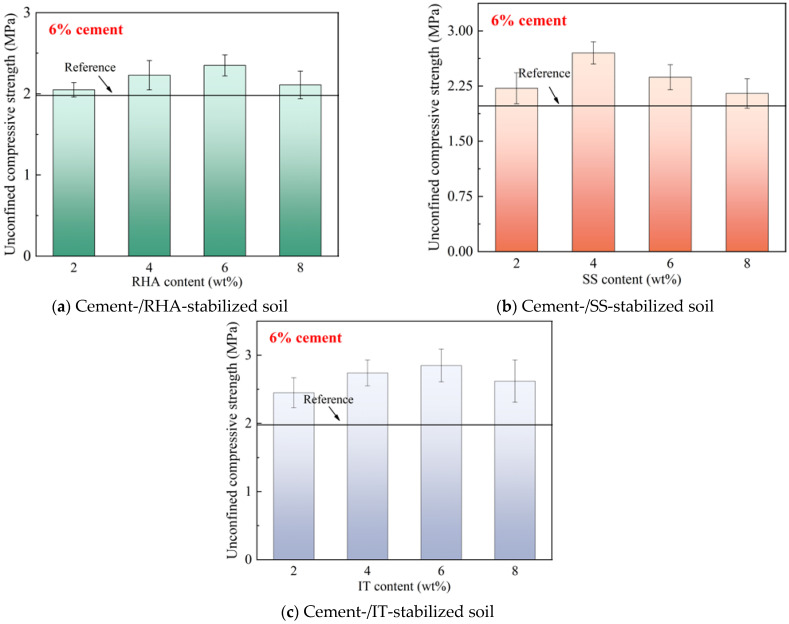
28-day unconfined compressive strength of stabilized soil with 6% cement content.

Figure 3 further depicts the 28-day unconfined compressive strength of the stabilized soil samples. It should be noted that, at such high cement contents, the mechanical strengths of the samples were much improved, and all the samples containing selected common waste materials showed higher strengths compared to the reference batches, indicating the positive effect of incorporation of RHA, SS and IT on improving the basic mechanical strength for stabilized soils. The RHA- and IT-blended samples showed the highest unconfined compressive strength with a waste material content of 6%, which became 4% for SS-stabilized cement soil. The enhanced hydration reaction may further promote the reactive activity for the common waste materials inside the cement soil.

### 3.2. Strength Changing Ratios of Stabilized Soil Samples with Various Waste Contents

**Figure 4:**
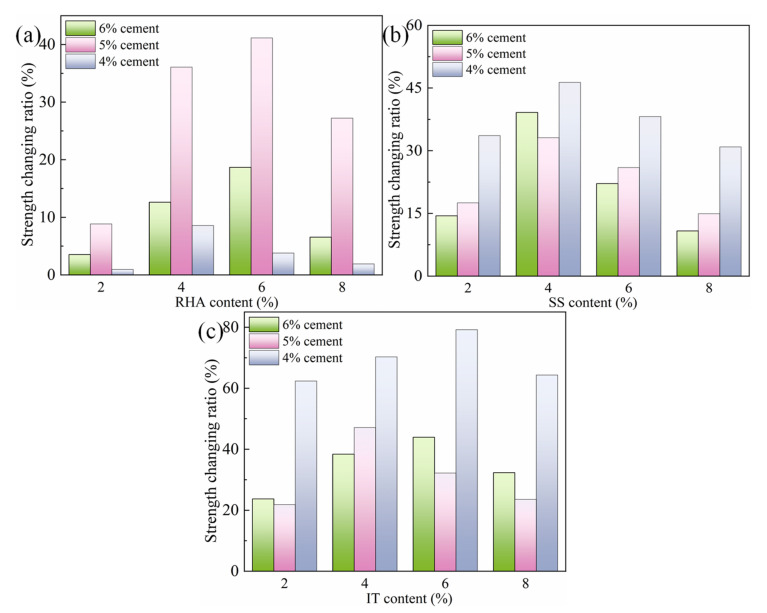
Strength changing ratios of stabilized soil containing various cement and common waste contents: (**a**) RHA, (**b**) SS and (**c**) IT.

As shown and demonstrated in the previous section, the 28-day unconfined compressive strength was used as the indicator to evaluate the effect of cement and the selected waste materials on the mechanical strength of stabilized soil samples. In this section, the effects of disparate variables will be discussed in detail. The inclusion of selected common waste materials increased the 28-day unconfined compressive strengths of stabilized soil samples. Figure 4 depicts the strength changing ratios of the samples including various contents of the common waste materials. As for RHA-blended samples, the improvement effect of RHA was not remarkable when the cement content was 4%, and the highest increasing ratio was lower than 10%, reflecting the poor effect of RHA. As the cement content increased, the influence of RHA became more significant and the strength increasing ratio reached up to 40% in the sample containing 5% cement with 6% RHA. Moreover, SS and IT both facilitated the strength formation of stabilized soil samples. Differing from RHA, the SS- and IT-blended samples showed the highest strength changing ratios when the cement content was 4%, which became 45% for the SS-incorporating sample and 78% for the IT-blended sample. As the cement content increased, the improvement effect of SS and IT was not always high but was still maintained at a satisfying level. The strength changing ratio was above 40% for SS-blended samples, with a cement content of 6% and SS content of 4%. As for the IT-blended samples, the strength changing ratios were always higher than 20%, highlighting the benefits of IT inclusion on the strength development of stabilized soil samples. Moreover, it should also be noted that the effect of cement on the compressive strength could be more significant, because the hydration reaction of cement was dominant in this material system. Under these circumstances, the following hydration analysis focuses on the performance of the IT-blended samples containing 6% cement content.

### 3.3. Hydration Degree of Stabilized Soil Samples

**Figure 5:**
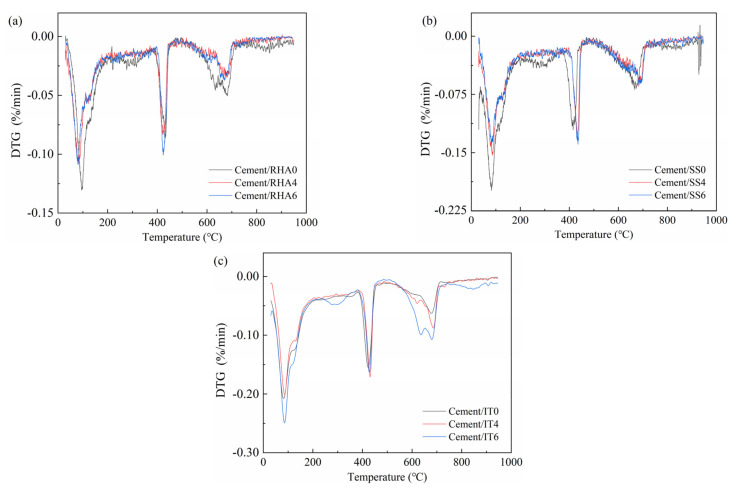
DTG curves of stabilized soil with 6% cement content and various common waste contents. (**a**) Cement-/RHA-stabilized soil; (**b**) Cement-/SS-stabilized soil; (**c**) Cement-/IT-stabilized soil.

DTG curves were used to evaluate the hydration degree of the stabilized soil samples, as depicted in Figure 5 [35,36]. In this section, the soil samples containing 6% cement were taken as the representative, and as illustrated in Section 3.2, the increasing cement contents contributed to improving the mechanical strength of the samples. When the cement content was 6%, the soil samples possessed the highest compressive strength, and thus this batch was used for further analysis. Moreover, the contents of the common waste materials were set to be 0%, 4% and 6%. In general, different characteristic peaks on the DTG curves indicate different composition phases inside the samples. To be specific, there are three main decomposition peaks shown on DTG curves. The peak shown within 200 °C indicates the decomposition of free water and the physically bonded water, the peak presented around 400 °C is connected with the dihydroxylation of Ca(OH)_2_ and the peak exhibited between 600–700 °C represents the decomposition of CaCO_3_ [37,38,39,40,41]. As depicted in Figure 5, the decomposition of the main hydration product Ca(OH)_2_ was more significant in SS- and IT-blended samples, indicating that more hydration products were generated in the samples, reflecting the more intense reaction inside the samples, which was beneficial to the strength improvement for the samples. However, the samples including RHA showed a much lower reaction reactivity with a less significant decomposition peak. The DTG results were consistent with the mechanical strength and highlighted the advantage of SS and IT inclusion on improving the hydration degree of stabilized soil samples. In addition, it needs to be stated that there were some organic contents in the sample, which were not mainly focused on in this work, because this section aims to investigate the hydration degree of cementitious materials in the stabilized samples.

### 3.4. Pore Structure of Stabilized Soil Samples

**Figure 6:**
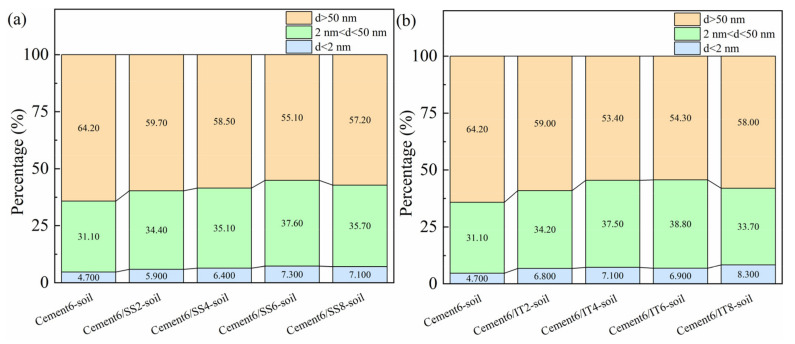
Accumulated pore volume for blended stabilized soil samples: (**a**) SS blended samples and (**b**) IT blended samples.

The pore structure analysis was further conducted based on a BET test, and the SS- and IT-blended samples with 6% cement addition were taken as the representative, because the enhanced effects of SS and IT on the mechanical strength were more remarkable. Figure 6 depicts the accumulated pore volume of the samples, and it can be found that the inclusion of SS and IT densified the pores of stabilized soil samples, and IT showed a more remarkable effect in this. Specifically, the large pores (with the pore size > 50 nm) took a dominant role in all the samples, which was reasonable as the insignificant hydration reaction [37,42]. However, the inclusion of SS and IT can lower the percentage of large pores and increase the percentage of small pores (with the size < 2 nm), indicating the densification effect of waste materials. Moreover, the percentages of large pores in SS-blended samples were always higher than those in IT-blended samples, highlighting the benefits of IT in densifying the pore structure. It should be noted that, when the IT content was 8%, the percentage of large pores increased, which can be explained by the fact that when the IT content was too high, this could hinder the hydration reaction inside the stabilized sample, and more large pores could exist. This phenomenon proves that a suitable content of waste material in stabilized soil is important to ensure the performance development. The pore structure analysis results further verified the advantage of IT in densifying the pore structure for the samples, which can explain the superior mechanical strength for the samples incorporating IT.

## 4. Conclusions

In this work, three kinds of common waste materials (RHA, SS and IT) were used as stabilizers with cement to enhance the performance of soil. Waste materials exhibited great ability in improving the mechanical strength and modifying the microstructure for the samples. To be specific, the selected waste materials showed different influences on the mechanical strengths of the stabilized soil samples. RHA showed the lowest reactivity with a much lower compressive strength compared to those of the SS- and IT-blended samples when the cement content was 4%. When the cement contents were 5% and 6%, the strength changing ratios of RHA blended samples increased, and the compressive strengths were much improved, especially for the IT- and SS-blended samples. To be more specific, the strength increasing ratios of SS-blended samples can reach up to 40%, which increased to 60% for IT-blended ones. Moreover, as the cement contents increased, the 28-day unconfined compressive strengths of the stabilized soil samples significantly improved, and the reaction activity of RHA also improved, indicating that the intense hydration reaction may promote the activity of waste material. The specific surface area test results can verify the effects of increasing cement contents on the hydration process of the soil samples. To be specific, the specific surface area was improved with the increasing cement contents, reflecting the intense hydration reaction inside the samples. In addition, the hydration degree and pore structure analyses further highlighted the benefits of SS and IT in improving the microstructure of the samples. The inclusion of waste materials enhanced the hydration degree, and more hydration products were found in SS- and IT-blended samples. Furthermore, SS and IT effectively densified the micro-pore structure of the samples—the percentages of large pores decreased with the aid of SS and IT incorporation.

## Figures and Tables

**Table 1:** Physical properties of the soil.

Soil Type	Liquid Limit, WL/%	Plastic Limit, WP/%	Plastic Index, IP	Optimum Moisture Content/%	Maximum Dry Density/(kg/m^3^)
Soft soil	42.7	21.6	21.1	18	1741

**Table 2:** Chemical compositions of raw materials.

	SiO_2_	CaO	Al_2_O_3_	Fe_2_O_3_	MgO
Cement	21.05	64.38	6.17	3.87	1.85
RHA	87.79	0.74	1.16		0.48
SS	14.46	40.42	4.15	23.89	8.12
IT	64.36	3.15	14.87	13.46	3.85

## Data Availability

The data presented in this study are available on request from the corresponding author due to privacy.
